# Evaluation of Gastrointestinal Endoparasites in Cattle in Central Spain: Focus on *Calicophoron daubneyi* with Coprological, Epidemiological, and Anthelmintic Insights

**DOI:** 10.3390/pathogens14101057

**Published:** 2025-10-19

**Authors:** Nélida Fernández Pato, Óscar García Barrero, Elvira Rodríguez Velasco, Félix Valcárcel Sancho, Jaime Galán Elvira

**Affiliations:** 1 Facultad de Veterinaria, Universidad Alfonso X el Sabio (UAX), Villanueva de la Cañada, 28691 Madrid, Spain; jgalaelv@uax.es; 2 Independent Clinical Veterinarian, 40001 Segovia, Spain; garos60@gmail.com (Ó.G.B.); elvira_vet@hotmail.es (E.R.V.); 3 Parasitology Group, Animal Reproduction Department INIA-CSIC, 28040 Madrid, Spain; valcarcel.felix@inia.csic.es

**Keywords:** *Calicophoron daubneyi*, cattle health, prevalence, parasite dynamics, paramphistomosis, epidemiological survey

## Abstract

*Calicophoron daubneyi*, a rumen fluke increasingly reported in European livestock, has emerged as a relevant parasitic threat in cattle. This study investigated the prevalence and seasonal dynamics of gastrointestinal endoparasites in 382 fecal samples from 40 beef cattle farms (26 extensive and 14 semi-extensive) in central Spain. Samples were analyzed using flotation, sedimentation, and modified McMaster techniques, complemented by PCR confirmation of trematodes and a 25-variable epidemiological survey. *C. daubneyi* was detected in 38.74% of samples and 77.5% of farms, surpassing *Fasciola hepatica* (13.09%), gastrointestinal nematodes (42.15%), and *Eimeria* spp. (16.75%). Mixed infections were frequent. Seasonal shedding patterns varied by parasite, with *C. daubneyi* peaking in spring and winter. Statistical analyses (Kruskal–Wallis, ANOVA, Mann–Whitney U) revealed significant seasonal differences and confirmed higher *F. hepatica* egg counts in extensive systems (*p* = 0.0012). Anthelmintic treatment was infrequent and mainly guided by coprological diagnosis; ivermectin, closantel, albendazole, and nitroxinil were the most used drugs, though none fully effective against *C. daubneyi*. Anthelmintic resistance was not evaluated in this study. These findings confirm the emergence of *C. daubneyi* in central Spain and highlight the need for targeted surveillance and seasonally adjusted control strategies.

## 1. Introduction

Extensive and semi-extensive cattle farming systems increase the exposure of animals to environmental parasitic stages, favoring the transmission of a wide range of digestive endoparasites. The prevalence and impact of these parasites depend on environmental conditions, farm management practices, and host-related factors [1].

Among gastrointestinal parasites, gastrointestinal nematodes (GIN) are typically the most prevalent and economically significant [2]. *Fasciola hepatica* is also common in areas where its intermediate host, the freshwater snail *Galba truncatula*, is present [3]. However, in recent years, another platyhelminth, *Calicophoron daubneyi*, has gained attention due to its increasing detection across Europe [4]. In Spain, its presence has been confirmed in Galicia [5,6,7], Asturias, Cantabria, Basque Country and Castilla y León [8]. *C. daubneyi* is a digenetic trematode of the Family *Paramphistomidae*. Its adult stage inhabits the rumen and reticulum, where it can cause paramphistomosis, a disease associated with diarrhea, weight loss, and reduced productivity in cattle. The parasite has an indirect life cycle involving freshwater snails, particularly *Galba truncatula*, as intermediate hosts. Diagnosis is challenging due to the morphological similarity of its eggs with those of *F. hepatica*, requiring sedimentation methods and, increasingly, PCR confirmation. Its recent emergence in Europe has been linked to climatic and management factors, making it an important health and economic concern in ruminant production systems.

*C. daubneyi* has been reported in various domestic and wild ruminants, with the highest prevalence observed in cattle [9]. It has also been detected in sheep, goats [10], and wild species such as mouflon, fallow deer, red deer, roe deer, and sika deer [11,12,13]. Like *F. hepatica*, *C. daubneyi* uses *G. truncatula* as its intermediate host [9,14,15]. Although coinfections in snails are rare due to seasonal dynamics [14], mixed infections in definitive hosts have been documented both in vivo and post-mortem [5,11,16].

The increasing prevalence of *C. daubneyi* in Europe has been associated with multiple factors, including animal age [8], vegetation, rainfall, and soil type—particularly silty and clay soils that favor snail habitats [17]. Farm location, grazing in mountainous terrain, herd size, and production system (extensive vs. intensive) also influence parasite transmission [16,18].

Importantly, current anthelmintic protocols targeting *F. hepatica* show limited or no efficacy against *C. daubneyi*, facilitating its spread [19,20]. The limited efficacy of commonly used drugs such as albendazole and ivermectin against this parasite underscores the need for updated control strategies.

Despite previous studies, important gaps remain regarding the prevalence, seasonal excretion patterns, and co-infection dynamics of *C. daubneyi* in cattle, particularly in northwestern central Spain. Farm-level risk factors and effective control strategies are also poorly characterized.

The aim of this study was to assess the prevalence and seasonal excretion patterns of *C. daubneyi* in beef cattle, evaluate co-infection with other digestive helminths, and identify farm-level factors associated with infection risk in northwestern central Spain. This region was selected due to its unique combination of climatic conditions, livestock density, and historical reports of trematode infections, which make it a relevant area for studying the epidemiology of *C. daubneyi*. Through a combination of coprological analyses and an epidemiological survey, we explored the presence of this emerging parasite and the factors associated with its distribution, including farm management practices and anthelmintic use.

## 2. Materials and Methods

### 2.1. Study Area 

**Figure 1:**
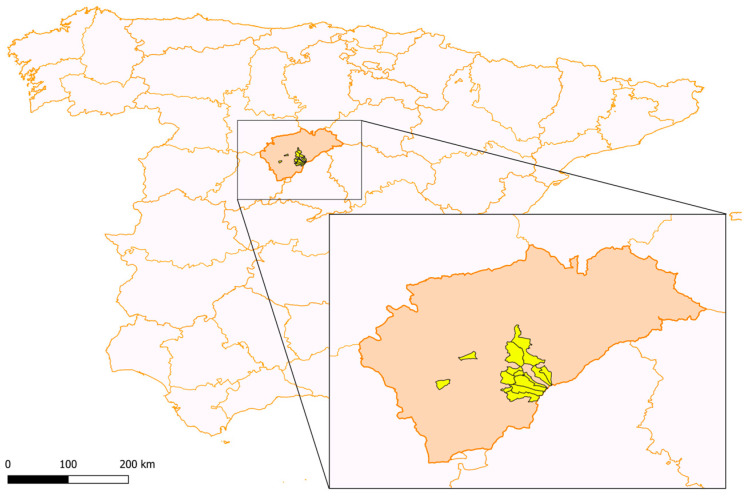
Map showing the distribution of the provinces of Spain, with the province of Segovia enlarged to highlight the geographical locations of the farms sampled.

This research was conducted in 40 cattle farms located in 24 different city councils of the south of Segovia province (Castilla y León, NW central Spain), covering an area of approximately 6920 km^2^. The study area is characterized by a temperate climate with dry summers (Csb), according to Köppen classification, with an average annual rainfall of around 500 mm [21]. Livestock density in the area is estimated at 35 cattle per km^2^. The geographical location of Segovia in Spain and of the farms that were included in the research are shown in Figure 1 and the geographic coordinates and administrative classification in Table 1.

### 2.2. Epidemiological Questionnaire 

Information was collected on (i) main characteristics of the farms, (ii) farm management practices, and (iii) use of drugs against helminths. The full questionnaire is provided in the Supplementary Materials.

### 2.3. Questionnaire Validation

The epidemiological questionnaire was designed to collect farm-level data on management practices, environmental conditions, and parasite control strategies. Operational definitions were established for key variables, including rotational grazing (periodic movement of cattle between pastures), anthelmintic treatment frequency, and wildlife presence (binary: yes/no).

The questionnaire was pilot tested in three farms not included in the final dataset to assess clarity and consistency. Minor adjustments were made to improve comprehension.

Missing data were handled as follows: non-critical variables were recorded as “unknown” and excluded from related analyses; farms with missing responses for critical variables were excluded from specific statistical models to avoid bias.

### 2.4. Parasite Collection and Processing 

Fecal samples were collected immediately after defecation directly from the ground. Approximately 200 g of feces were obtained per animal. Each sample therefore corresponded to a different individual, although individual identification was not possible. Material was taken from the center of the fecal mass to minimize contamination. 

All samples were collected individually and not pooled and taken from the central fecal mass to minimize environmental contamination. Samples were stored in gloves from which air was removed to preserve integrity. 

Sampling was conducted in 40 cattle farms located in southern Segovia. In spring, 15 farms were sampled, of which 9 were extensive and 6 semi-extensive. In summer, 4 farms were sampled, all of them extensive. In autumn, 15 farms were sampled, 14 of which were extensive and 1 semi-extensive. In winter, 20 farms were sampled, including 13 extensive and 7 semi-extensive.

A total of 382 fecal samples were collected across all seasons. Seasonal sample sizes were as follows: spring (n = 90), summer (n = 27), autumn (n = 77), and winter (n = 188). These values were considered in all seasonal comparisons and statistical analyses. On average, 10.05 fecal samples (±13.44 SD) were collected per farm. Samples were obtained in an average of 1.37 seasons (±0.59 SD) per farm.

They were stored at 5 °C until laboratory analysis was performed within 48 h after collection. Initially, a macroscopic analysis was conducted, followed by two microscopic analyses: flotation using a modified McMaster technique and sedimentation.

The modified McMaster flotation method was used to detect nematode eggs and *Eimeria* oocysts. Briefly, 5 g of feces were homogenized in 40 mL of saturated saline solution. The mixture was filtered and loaded into McMaster chambers, and eggs/oocysts were counted under a microscope. Due to the high density of trematode eggs, this method is not sensitive to detecting flukes such as *F. hepatica* or *C. daubneyi*. The sedimentation technique was conducted with 20 g of feces, and in this case, the entire sediment was analyzed. Endoparasites were identified according to previous descriptions [22,23,24].

Trematode eggs obtained through the fecal sedimentation technique were centrifuged to concentrate them. Subsequently, under an inverted microscope, the eggs were separated by color (yellow and colorless) and kept in distilled water at refrigeration temperature (5 °C) until the molecular technique was performed.

Following coprological techniques, some individuals appeared to be infected by *C. daubneyi*. A total of 40 adult trematodes were collected from the rumen and reticulum of five cattle from these farms at a slaughterhouse in Segovia. Subsequently, the trematodes were cut into small pieces and stored at −80 °C until DNA extraction.

### 2.5. PCR Identification 

DNA extraction (from colorless eggs and from adults) and PCR amplification were performed according Martínez-Ibeas et al. [25]. Briefly, adult flukes and eggs of *F. hepatica* and *C. daubneyi* were lysed by adding 1 mL of TRIzol™ Reagent Reagent (Invitrogen^®^, Carñsbd, CA, USA) per 50–100 mg of tissue to the samples and were frozen at −80 °C. Samples were introduced into a metal cylinder submerged in liquid nitrogen and were ground thoroughly with a pestle until they were completely pulverized. After incubation at room temperature for 5 min, 0.2 mL of chloroform was added, followed by a 3-min incubation. Samples were centrifuged at 12,000 *g* for 15 min at 4 °C. The aqueous phase was discarded. 

DNA purification was performed using the commercial QIAamp^®^ DNA Mini Kit (Qiagen, Hilden, Germany), following the instructions of the manufacturer.

DNA purity (260/280 nm absorbance ratio) and concentration were assessed using a NanoDrop spectrophotometer (Thermo Scientific, Wartham, MA, USA).

Purified DNA samples were subjected to PCR amplification using primers targeting the cytochrome c oxidase subunit 1 (cox1) gene of *Calicophoron daubneyi*, as follows: forward primer 5′-GTTTGTGTGGTTTGCCACGG-3′ and reverse primer 5′-CTACCCCAAGCAGCCACTAC-3′ [18]. The reaction was carried out in a final volume of 20 μL, containing 10 μL of Supreme NZYTaq II 2× Green Master Mix (NZYTech), 10 ng of template DNA, and 4 μL of 10 μM specific primers. PCR conditions included an initial denaturation at 92 °C for 2 min, followed by 38 cycles of denaturation at 95 °C for 30 s, annealing at 65 °C for 30 s, and extension at 72 °C for 1.5 min. A final extension step was performed at 72 °C for 10 min. PCR products were visualized by electrophoresis on 1.5% agarose gels stained with GelRed, ran at 120 V for 30 min, and photographed using a transilluminator imaging system. The expected amplicon size was 169 bp.

### 2.6. Statistical Analysis

Seasonal comparisons of parasitic load were performed using both parametric and non-parametric tests depending on the distribution of the data. Specifically, ANOVA was applied when normality assumptions were met or approximately satisfied, and Kruskal–Wallis tests were used as a non-parametric alternative when data did not meet normality criteria. A total of 33 seasonal comparisons were conducted across five parasite taxa. The use of ANOVA implies that normality assumptions were considered, and the inclusion of Kruskal–Wallis tests further supports the robustness of the analysis. All statistical analyses were performed using appropriate software, and significance was considered at *p* < 0.05.

## 3. Results

### 3.1. Epidemiological Survey

#### 3.1.1. Description of Farms: Animal Factors and Management

A total of 40 beef cattle farms (26 extensive and 14 semi-extensive) were included in the present study. All farms were dedicated to meat production. In 31 farms, cattle were the only animal species present, while in the remaining nine, cattle shared pastures with wild roe deer, sheep, and/or horses. The average number of males per farm was 19.42 (SD ± 18.53; range: 1–110), and for females, the average was 96.31 (SD ± 89.97; range: 20–550). A wide variety of cattle breeds was observed, with the Avileña-Black Iberian cattle breed (purebred or crossbred) being the most frequent, followed by Limousine, and less frequently Charolais, Asturian, Tudanca, and Fleckvieh. Pasture rotation was practiced in 90.48% of extensive farms and 35.71% of semi-extensive farms. The remaining general characteristics of the farms are summarized in Table 2.

#### 3.1.2. Farm Deworming Procedures 

Deworming was reported in 2 out of 11 extensive farms (18.18%) and in none of the semi-intensive farms surveyed. Treatments were applied sporadically, with the last deworming occurring between 1 and 8 years prior to the study, and no consistent seasonal pattern was observed. In extensive systems, the decision to deworm was primarily based on coprological analysis (63.64%), followed by farmer decision and veterinary advice (18.18% each). In contrast, in semi-intensive systems, coprological results were less frequently used (27.27%), while farmer decision and veterinary advice were each cited in 36.36% of cases. The most used anthelmintics belonged to three main classes: salicylanilides (nitroxinil and closantel), macrocyclic lactones (ivermectin), and benzimidazoles (albendazole), as detailed in Table 3. Percentages represent the proportion of farms within each production system reporting the respective practices.

### 3.2. Coprological Results 

#### 3.2.1. Total Collected Fecal Samples 

Seven different digestive endoparasites were identified in the collected samples. Gastrointestinal nematodes (GIN) were detected in 42.15% of the samples, followed by *C. daubneyi* (38.74%), *Eimeria* spp. (16.75%), *F. hepatica* (13.09%), *Buxtonella sulcata* (1.31%), *Dicrocoelium dendriticum* (0.79%), *Trichuris discolor* (0.26%), and *Moniezia benedeni* (0.26%). These findings are summarized in Table 4.

#### 3.2.2. Total Collected Fecal Samples and Season

**Figure 2:**
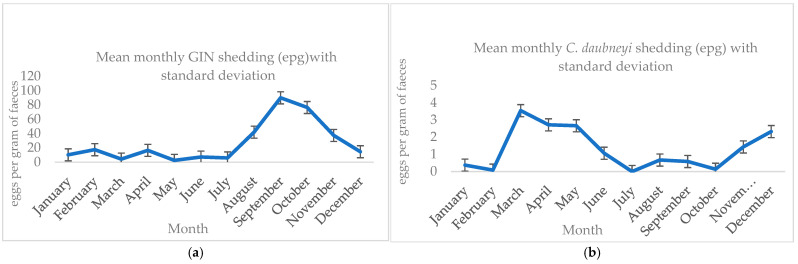

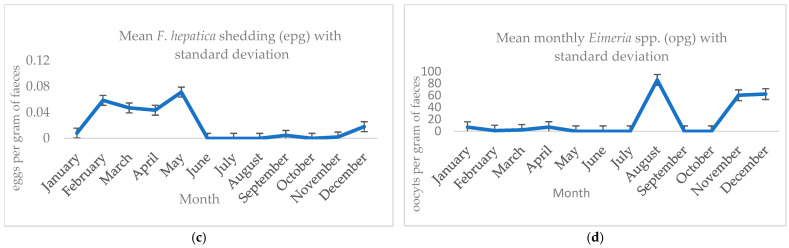
Monthly mean excretion patterns of the main parasites, including standard deviation, based on quantitative coprological analysis: eggs per gram of feces (EPG) for gastrointestinal nematodes, *C. daubneyi* and *F. hepatica*; oocysts per gram of feces (OPG) for *Eimeria* spp.). (**a**) GIN epg; (**b**) *C. daubneyi* epg (**c**); *F. hepatica* epg and (**d**) *Eimeria* spp. opg.

The fecal counts (FC) varied depending on the parasite and the season. The highest FC was observed during the summer and autumn for *Eimeria* spp., followed by GIN during the autumn, as shown in Table 5. The excretion pattern of *C. daubneyi* was higher in spring and winter, while *F. hepatica* showed a similar pattern but with a much lower mean excretion throughout the year (Figure 2).

Patent infections by *C. daubneyi* were detected in 38.74% of samples, whereas *F. hepatica* was found in 13.09%. GIN alone were identified in 16.49% of samples, while *Eimeria* spp. alone were present in 3.4% of samples.

Mixed infections were nearly as frequent as single-parasite infections, being observed in 49.19% of the samples analyzed. Co-infections involving *C. daubneyi* were recorded with all gastrointestinal parasites detected in the research. Specifically, double infections were found in 15.45% of samples, triple infections in 6.54%, and quadruple infections in 1.04% of samples, as detailed in Table 5 with co-infections and farms.

#### 3.2.3. Coprological Results Considering Total Number of Farms 

Digestive endoparasites were detected in 100% of the cattle farms using various coprological techniques. GIN were found in 85% of the farms, while *T. discolor* was detected in only one farm (2.5%). GIN were the most prevalent and environmentally impactful parasites. Notably, ten fecal samples exhibited egg counts exceeding 200 epg, of which 70% originated from extensive farming systems. Regarding seasonality, 60% of these high FEC results were recorded in autumn, 20% in winter, and 10% each in summer and spring.

Trematodes were present in 80% of the farms, with *C. daubneyi* (77.50%), *F. hepatica* (32.50%), and *D. dendriticum* (7.50%) identified. *Eimeria* spp. oocysts, *B. sulcata* cysts, and *Moniezia* eggs were found in 40.00%, 10.00%, and 2.50% of the farms, respectively.

Patent infections by *C. daubneyi* were detected in 77.5% of farms and 38.74, *F. hepatica in* 35.0%, GIN alone were identified in 52.5%, while *Eimeria* spp. in 22.5% of farms.

Co-infections were recorded as mentioned previously. Double infections were found in 60% of farms, triple infections in 17.5% and quadruple infections in 12.5% of farms. As shown in Table 6.

#### 3.2.4. Coprological Results Considering Total Number of Farms and Season

The number of coprological positive results varied seasonally. The highest number of positive farms was observed in winter for GIN and in spring for *C. daubneyi* (32.5% each). This was followed by 27.50% of farms in winter and autumn for *C. daubneyi* and in spring for GIN. The lowest number of positive farms was recorded in summer: 10% for GIN, 5% for *C. daubneyi*, 2.50% positive for *Eimeria* spp., and none for *F. hepatica*.

The parasitism rates of the main digestive endoparasites: GIN, *C. daubneyi*, *F. hepatica* y *Eimeria* spp., were also calculated according to the production system (extensive or semi-intensive) as detailed in Table 7. 

### 3.3. Statistical Analysis of Digestive Endoparasite Detection

#### 3.3.1. Seasonal Differences in Parasite Excretion

Statistical analyses were conducted to evaluate seasonal differences in the elimination of the most prevalent digestive endoparasites using Kruskal–Wallis and ANOVA tests. Statistically significant differences were detected for *Eimeria* spp. with both tests. Kruskal–Wallis also revealed significant differences for *C. daubneyi* and *F. hepatica*, while ANOVA showed significance for *Eimeria* spp. and gastrointestinal nematodes (GIN). *C. daubneyi* was close to the significance threshold in ANOVA (*p* > 0.05). These results are summarized in Table 6.

To compare *F. hepatica* fecal egg counts (FEC) between cattle raised under extensive and semi-extensive production systems, we first assessed the normality of the data using the Shapiro–Wilk test, which indicated that FEC values did not follow a normal distribution in either group (*p* < 0.001). Consequently, we applied the non-parametric Mann–Whitney U test to evaluate differences between groups. Statistical analyses were performed using R v.4.2.2.

Statistically significant differences were observed in the fecal egg counts (FEC) of *F. hepatica* between extensive and semi-extensive production systems (Mann–Whitney U = 12759.0, *p* = 0.0012), with higher elimination in extensive systems.

Statistical analyses were conducted to evaluate seasonal differences in the detection of the most prevalent digestive endoparasites using Kruskal–Wallis and ANOVA tests. Statistically significant differences were detected for *Eimeria* spp. with both tests, detailed in Table 8. Kruskal–Wallis also revealed significant differences for *C. daubneyi* and *F. hepatica*, while ANOVA showed significance for *Eimeria* spp. and GIN. *C. daubneyi* was close to the significance threshold in ANOVA (*p* > 0.05).

The Fisher Test was applied to assess the independence between the presence of both parasites. The test yielded a *p*-value of 0.00004, indicating a statistically significant association between the detection of *F. hepatica* and *C. daubneyi* (*p* < 0.05).

This contingency analysis was performed to evaluate the association between coprological detections of *F. hepatica* and *C. daubneyi*. The results are summarized in Table 9.

#### 3.3.2. Seasonal Significance by Parasite

Seasonal variation in parasite shedding was assessed using both Kruskal–Wallis and ANOVA tests. Statistically significant differences were observed for *C. daubneyi* in spring, for GIN and *Eimeria* spp. in winter, and additionally for *Eimeria* spp. in autumn. The results are summarized in Table 10.

#### 3.3.3. Differences According to Production System

Statistical comparisons were made between extensive and semi-extensive farming systems using the Mann–Whitney U test. Significant differences in *F. hepatica* egg counts between production systems were confirmed using (*p* = 0.0423), due to non-normal distribution and unequal variances. Extensive systems showed significantly higher egg elimination.

#### 3.3.4. Differences Between Breeds

No significant differences were found between breeds in the fecal parasite detection of *T. discolor*, *D. dendriticum*, and *B. sulcata*. For the remaining parasites, some differences were observed between specific breeds, although not all were statistically significant. The statistical analyses corresponding to these latter gastrointestinal parasites are available in the Supplementary Materials.

### 3.4. PCR Results

Adult trematodes recovered from the rumen and reticulum of cattle were identified as *Calicophoron daubneyi* based on their typical external morphology: conical rather than flat, pear-shaped, pink-colored, measuring 0.5–1.0 cm in length, and possessing two suckers, with the acetabulum located posteriorly. 

**Figure 3:**
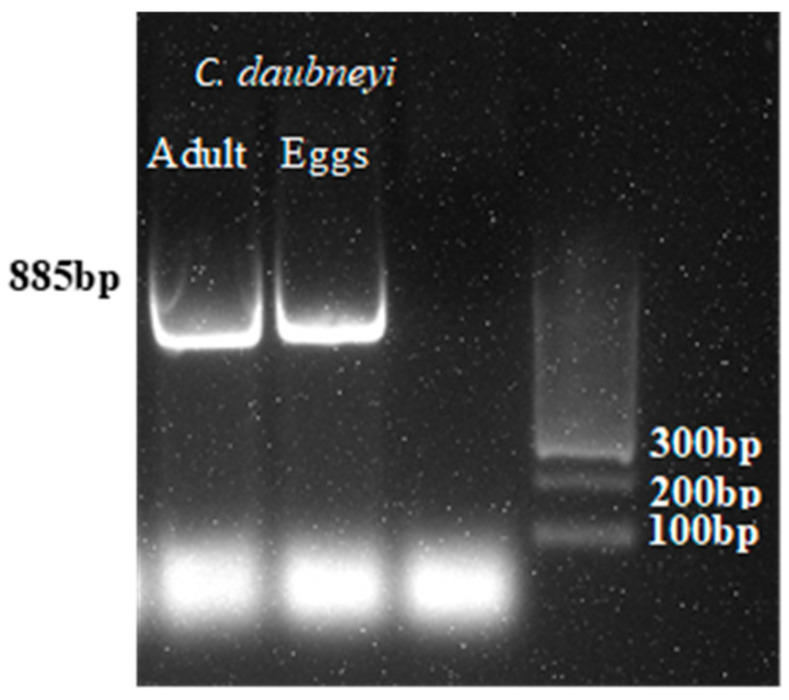
Products of PCR amplification of *C. daubneyi* adults and eggs in agarose with Gel Red. *C. daubneyi* adult, *C. daubneyi* egg negative control. Low-range DNA ladder (100–300 bp) was used for fragment size estimation.

The specific amplification of *C. daubneyi* was detected in both adults and eggs, as shown in Figure 3, confirming the morphological identification.

## 4. Discussion

Infection by digestive endoparasites in cattle raised under extensive or semi-extensive production systems is a well-established phenomenon, as demonstrated in this study. Coprological analyses revealed that 100% of the surveyed farms tested positive for at least one parasitic species. However, it is important to emphasize that relying on a single coprological method may be insufficient, as not all techniques exhibit equal sensitivity for detecting the most prevalent parasites in each host species [26].

Gastrointestinal nematodes (GIN), representing a group of nematode species, were the most frequently detected endoparasites, with 85% of farms testing positive. These results are consistent with previous studies, both in terms of overall prevalence and sample-based detection rates [27] and are notably higher than those reported in other investigations, where global prevalence did not exceed 39% [28]. Nevertheless, only six samples exhibited fecal egg counts (FEC) above 200 eggs per gram (epg), highlighting that while GIN are cosmopolitan and widely distributed in cattle farms in central Spain, FEC alone may not fully reflect the actual health impact. Exceptions may exist in specific cases where selective control measures are warranted. It is also important to consider that adult animals, due to acquired resistance mechanisms against GIN infection, typically exhibit lower FEC values compared to younger individuals [29].

The second most prevalent digestive endoparasite identified in this study was *C. daubneyi*, with an individual sample prevalence of 38.74% and presence in 80% of the farms surveyed. These figures exceed those reported in other regions of Spain, such as Galicia, where individual prevalence was 19% and herd-level prevalence reached 36% [30]. These figures exceed those reported in other regions of Spain, such as Galicia, where individual prevalence was 19% and herd-level prevalence reached 36% [30]. However, they are still lower than the most recent estimates of 45.6% reported in a preprint by García-Dios, 2025 [31]. Similarly, a study conducted in Italy reported coprological prevalence rates of 55% at the farm level. Although specific prevalence data are not always provided, the high occurrence of *C. daubneyi* in cattle has also been highlighted in the United Kingdom and Western Europe [32]. 

Regarding *Eimeria* spp., our findings differ from those of other studies in which these coccidia were the most prevalent parasites, followed by GIN [33]. Age of sampled animals could not be reliably determined for most samples; thus, age-related interpretations are limited. The overall prevalence of *Eimeria* spp. was 16.75%, substantially lower than the 86.4% reported in Italian cattle farms [34]. This difference may be explained by the absence of herds with a recurrent history of coccidiosis in the present investigation.

The coprological global prevalence of *F. hepatica* was 13.09%, which falls within the wide range reported in Europe (0.1% to 86%) [35]. The FEC of this parasite is considered unreliable due to irregular egg shedding. Although coprological diagnosis can be useful for herd-level monitoring, its sensitivity is limited, particularly in early or subclinical infections [35,36]. Moreover, FEC values may be influenced by multiple factors, including host immunity, parasite biology and sampling timing, so their interpretation should be approached with caution, especially when used in isolation to infer infection intensity or compare production systems. Higher prevalence in farms with continuous grazing can be explained by increased exposure to infected pastures, highlighting the importance of rotational grazing as a control measure.

### 4.1. Coprological Techniques for the Detection of Digestive Endoparasites

The findings of this study confirm the utility of coprological techniques for detecting digestive endoparasites in cattle. However, it is recommended to employ a combination of diagnostic methods, including flotation and sedimentation techniques [26], as the most used routine method—modified McMaster—is unable to detect trematodes due to the high specific gravity of their eggs [37].

As previously mentioned, coprological analysis revealed *F. hepatica* eggs in 13.09% of samples and in 37.5% of farms, whereas *C. daubneyi* was detected in 38.74% of samples and 77.5% of farms. The prevalence and egg counts per gram (EPG) for *C. daubneyi* were consistently higher than those for *F. hepatica,* in agreement with previous studies [38,39]. Notably, co-infected cattle have shown EPG values up to ten times higher for *C. daubneyi* [18].

Moreover, the sensitivity of coprological techniques must be considered. As previously mentioned, these methods exhibit low sensitivity for detecting *F. hepatica*, although they appear to be more effective for identifying infections caused by *C. daubneyi*, as evidenced by previous studies and the present investigation, which reported 80% of farms positive for this parasite [36,37].

### 4.2. Epidemiological Factors

Due to the collection of fecal samples from the environment rather than directly from individual animals, age and sex could not be reliably assigned. Therefore, associations between prevalence and age or sex could not be assessed. These findings contrast with previous reports on *C. daubneyi*, which have documented age-related increases in infection rates and a higher prevalence among females [8]. This factor is particularly relevant, as the presence of parasitized adult animals contributes to pasture contamination and perpetuation of the life cycle of the parasite, a phenomenon previously emphasized [6].

Breed-related differences in fecal parasite excretion were generally limited across the livestock populations surveyed. No statistically significant variations were observed among breeds in the excretion of *T. discolor*, *D. dendriticum*, and *B. sulcata*. For other parasitic taxa, slight breed-associated trends were noted, although these were not consistent. For instance, the Fleickview breed exhibited significantly higher fecal egg counts (FEC) of GIN compared to other breeds, while the Tudanca breed showed lower GIN excretion but comparatively higher FECs of *C. daubneyi* and *Eimeria* spp. These differences, however, did not reach statistical significance when compared to other breeds or their crossbreeds.

Notably, the detection of *C. daubneyi* was associated with statistically significant differences in mixed Tudanca, mixed Avileña-Black Iberian cattle, Limousine–Avileña-Black Iberian cattle, and Limousine–Charolaise breeds. Similarly, *F. hepatica* prevalence showed significant variation in Avileña-Black Iberian cattle and Avileña-Black Iberian cattle–Limousine breeds. These findings suggest a potential influence of breed on susceptibility to certain trematode infections. However, it is important to consider that other environmental and management-related factors—such as pasture contamination levels and grazing duration—may also play a critical role in shaping parasite transmission dynamics and infection intensity, as previously noted [5].

Further studies integrating host genetics, grazing behavior, and pasture ecology are warranted to better understand the multifactorial nature of parasitic infections in livestock.

### 4.3. Production System

Throughout the study, the overall parasitism rate was higher in cattle raised under extensive production systems compared to those in semi-extensive systems (Table 5). This supports the notion that, when infective stages are present in the environment, increased exposure time—such as longer grazing hours—correlates with a higher probability of infection. It should be noted, however, that the number of samples collected from extensive systems was greater than that from semi-extensive systems.

Despite the higher detection rates in animals raised under extensive conditions, statistical comparisons were not performed for *F. hepatica* FEC due to the irregular egg shedding pattern of this parasite. Additionally, seasonal variation was evident in the most prevalent parasites identified in this study. Significantly higher excretion of *C. daubneyi* was observed in spring, while GIN and *Eimeria* spp. showed peak excretion in winter, with *Eimeria* spp. also presenting a secondary peak in autumn. FEC of *F. hepatica* was not considered due to its unreliable shedding pattern.

### 4.4. Epidemiological Insights: C. daubneyi

The results reported for *C. daubneyi* in this study differ from previous investigations conducted in Spain. Coprological studies have reported peak egg excretion of *C. daubneyi* during autumn and spring [11,30], while *post-mortem* examinations have also indicated a higher prevalence of this paramphistome in autumn [5]. These discrepancies may be explained by climatic differences between the regions studied, as northern Spain is generally more humid, favoring the persistence of trematodes such as *F. hepatica* and *C. daubneyi* throughout more months of the year.

Both *F. hepatica* and *C. daubneyi* share part of their life cycle, with the aquatic snail *G. truncatula* identified as the principal intermediate host for both species [10]. This shared biology has important epidemiological implications, as environmental factors such as temperature and rainfall directly influence the completion of their life cycles [18]. These conditions also facilitate mixed infections, as observed in the present study, where 8.6% of samples and 30% of farms showed co-infection with both trematodes. Mixed infections were more frequent in winter (3.9% of samples) and spring (15% of farms), while no cases were detected during summer.

The prevalence of mixed infections in this study is comparable to findings from other European regions, although slightly lower than the 46% reported in Wales [18], and below the 20% observed in northern Spain (Galicia) and Portugal [5]. However, it exceeds the 2.1% reported in Germany [13].

These differences may be attributed to the dry summer conditions in the studied farms, where pastures do not remain moist as they do in northern Spain. The only areas where water puddles can be observed—and where the life cycle of *F. hepatica* and *C. daubneyi* can be completed—are watering points, which likely serve as the main source of infection during the dry months of this season. 

In addition, a statistically significant association between both parasites was observed, suggesting that co-infections involving *C. daubneyi* and *F. hepatica* are likely, as reported previously. 

Regarding the other detected trematode species, further research is needed to clarify potential associations, given their low prevalence in the present study.

Coprological detection rates of *C. daubneyi* in central Spain reached 77.5% at the farm level and 38.79% at the individual sample level, confirming its presence and establishment in the study area. 

Similarly, *F. hepatica* remains a common parasite in cattle, as reflected in the anthelmintic protocols applied. In extensive systems, 80% of the treatments used were reported to be effective against *F. hepatica* based on published efficacy studies [19,40]; resistance was not directly evaluated in this study. Treatments included 20% albendazole, 20% closantel, and 40% nitroxinil. In semi-extensive systems, albendazole, closantel, and a combination of closantel with ivermectin were each used in 14.29% of cases.

Traditionally, many commonly used anthelmintics, including some of those mentioned, were considered ineffective against *C. daubneyi* [19]. However, recent studies have demonstrated variable efficacy of albendazole, clorsulon, closantel, and oxyclozanide, with reported effectiveness ranging from 62% to 80% in naturally infected animals [20]. Among these, oxyclozanide has historically been regarded as the most effective treatment against *C. daubneyi* [19] and recent research has reported complete efficacy (100%) in the treatment of adult flukes [40].

Although some active compounds used for deworming show partial efficacy against *C. daubneyi*, treatments are typically administered only in spring in extensive systems, and in spring or both spring and autumn in semi-extensive systems. This contrasts with the seasonal pattern observed in the present study, where the highest fecal egg counts of *C. daubneyi* were recorded in winter.

These findings support the consideration of adjusting the timing of anthelmintic treatments in farms where *C. daubneyi* and *F. hepatica* are present. This is particularly relevant for extensive production systems, which show higher prevalence and environmental contamination. It may be advisable to advance the deworming schedule to before spring, to reduce pasture contamination and interrupt the seasonal transmission cycle.

The increased detection of *C. daubneyi* appears to be multifactorial, potentially influenced by treatment protocols, environmental conditions, and the biology of the parasite. Notably, *C. daubneyi* has been shown to produce higher egg outputs than *F. hepatica*, which may contribute to its apparent dominance in coprological surveys [31,32]. In this study, patent infections of *C. daubneyi* were detected in all seasons, whereas *F. hepatica* was not observed during summer.

However, the absence of reports regarding its presence in certain regions with climates favorable to this parasite may be attributed to the lack of sedimentation-based coprological methods that enable its identification, or to the failure to recognize its eggs in fecal samples. Although its eggs can be distinguished from those of *F. hepatica* by their lack of yellowish pigmentation, a non-expert observer might still misidentify them [41].

To the best of our knowledge, no previous studies have reported coprological detection or seasonal patterns of *C. daubneyi* in the central region of Spain. This lack of prior data underscores the novelty and epidemiological relevance of our findings, particularly in a region where fasciolosis has traditionally received more attention. When considered as a whole, these results support the hypothesis that *C. daubneyi* is an emerging parasite in Europe [15,16]. Further epidemiological studies are needed to better understand its impact and to develop control strategies that consider its specific transmission dynamics and ecological requirements.

### 4.5. Study Limitations

Limitations of this study include non-random sampling, collection of feces from the environment rather than directly from individual animals, and the potential for biased prevalence estimates. One of the criteria during sampling was spontaneous defecation to avoid repeated collection from the same individual, which may affect sample representativeness. Additionally, animals were grazing freely during field visits, and it was not possible to approach them to verify identification numbers, preventing individual-level data collection. Fieldwork conditions also limit the number of samples and the ability to apply strict randomization. Therefore, results regarding prevalence and associated factors should be interpreted with caution. Future studies should consider more systematic sampling strategies, individual fecal collection when feasible, and expanded molecular and geospatial analyses to strengthen findings.

## 5. Conclusions

This study highlights the widespread presence of *C. daubneyi* in beef cattle farms in northwestern central Spain, confirming its status as an emerging parasite in Europe. The high prevalence, seasonal shedding patterns, and limited treatment options underscore the need to implement targeted surveillance and control strategies. While gastrointestinal nematodes and *Eimeria* spp. remain frequent, the increasing incidence of *C. daubneyi* requires further attention in parasitological monitoring and the development of effective anthelmintic protocols that should be based on epidemiological knowledge of each geographic region.

## Figures and Tables

**Table 1:** Geographic coordinates and administrative classification of cattle farm localities in the province of Segovia (Spain) during the coprological study.

Town	Latitude	Longitude	Administrative Classification
Adrada	41.3678	−4.1079	Municipality
Aldeasaz	41.0753	−3.9572	Neighborhood/Hamlet
Basardilla	41.0274	−4.026	Municipality
Berrocal	41.067	−3.967	Neighborhood/Hamlet
Brieva	41.0344	−4.0533	Municipality
Collado Hermoso	41.0386	−3.9191	Municipality
Espirdo	40.9976	−4.0733	Municipality
Fuentemilanos	40.8749	−4.2277	Neighborhood
La Cuesta	41.0828	−3.9602	Hamlet
Losana del Pirón	41.0678	−4.0228	Minor Local Entity
Madrona	40.9333	−4.1333	Neighborhood
Marazuela	41.1686	−4.4222	Municipality
Palazuelos	40.9167	−4.0667	Municipality
Pelayos	41.0931	−3.9814	Municipality
Revenga	40.9167	−4.1167	Neighborhood
San Cristobal	40.95	−4.1	Municipality
San Ildefonso	40.9014	−4.0036	Municipality
Sotosalbos	40.9833	−3.9833	Municipality
Tabanera	41	−4.0833	Municipality
Tizneros	41.0167	−4.0667	Hamlet
Torrecaballeros	40.9833	−4.0333	Municipality
Torreiglesias	41.0833	−4.0167	Municipality
Tres Casas	40.95	−4.1	Neighborhood
Valsaín	40.9	−4.0167	Minor Local Entity

**Table 2:** Main descriptors of cattle farms in northwestern central Spain. Numbers in parentheses indicate the number of valid responses obtained in the questionnaire. Percentages represent the proportion of farms corresponding to each category. For census data, values indicate the mean number of animals per farm, along with the minimum and maximum values.

Main Descriptors of Cattle Farms	Extensive	Semi-Extensive	Total
Animal species	(n = 26)	(n = 14)	(n = 40)
Cattle	69.23	92.86	77.50
Cattle/roe deer	19.23	7.14	15.00
Cattle/dog/roe deer	3.85		2.50
Cattle/dog/ sheep/ roe deer	3.85		2.50
Cattle/horses/roe deer	3.85		2.50
Aptitude: meat	100	100	100
Census (mean, ± SD)	(n = 25)	(n = 14)	(n = 39)
Cattle	132.88 ± 110.45	83.71 ± 52.33	115.23 ± 102.67
Males	22.58 ± 18.53	14.00 ± 12.34	19.42 ± 18.53
Females	111.20 ± 89.97	69.71 ± 45.12	96.31 ± 89.97
Age	(n = 23)	(n = 14)	(n = 39)
<6 months–2 years	8.7	7.14	7.69
<6 months–3 years	69.57	57.14	61.54
11 months–3 years		7.14	2.56
6 months–3 years		7.14	2.56
7 months–3 years		7.14	2.56
adults	21.74	14.29	17.95
Breed	(n = 26)	(n = 14)	(n = 40)
Avileña-Black Iberian cattle	3.85	0.00	2.50
Avileña-Black Iberian cattle/Charolais	3.85	14.29	7.50
Avileña-Black Iberian cattle/Limousine	7.69	7.14	7.50
Charolais		7.14	2.50
Charolais/mixed		7.14	2.50
Fleickview	3.85		2.50
Limousine	3.85	21.43	10.00
Mixed	53.85		35.00
Mixed/Avileña-Black Iberian cattle/Limousine		7.14	2.50
Mixed/Limousine	15.38	21.43	17.50
Mixed/Limousine/Asturian		7.14	2.50
Tudanca	3.85	7.14	5.00
Tudanca/Limousine	3.85		2.50
Management	(n = 23)	(n = 14)	(n = 37)
Age separation	39.13	28.57	35.14
Preweaning separation	43.48	14.29	32.43
Pasture property	78.26	35.71	62.16
Complete productive system	52.17	28.57	43.24
Partial productive system	47.83	71.43	56.76
	(n = 21)	(n = 14)	(n = 35)
Pasture rotation	90.48	35.71	68.37
Dry grass	85.71	100.00	91.43
Dry and wet grass	14.29	0.00	8.57

**Table 3:** Deworming practices reported in extensive and semi-intensive cattle farms in northwestern central Spain during coprological and epidemiological surveys. Percentages represent the proportion of farms using each practice or treatment. Values in parentheses indicate the number of valid responses.

Variable	Extensive	Semi-Extensive
Annual deworming	(n = 11)	(n = 2)
	18.18	0.00
Season of deworming	(n = 3)	(n = 9)
spring	100	44.44
summer		
autumn		33.33
winter		
spring/autumn		22.22
Anthelminthic class	(n = 5)	(n = 7)
albendazole	20.00	14.29
closantel	20.00	14.29
ivermectin	20.00	57.14
ivermectin + closantel		14.29
nitroxinil	40.00	
Drug selection	(n = 11)	(n = 11)
after coprology	63.64	27.27
farmer decision	18.18	36.36
vet advice	18.18	36.36

**Table 4:** Digestive endoparasites detected in 382 cattle fecal samples from beef farms of northwestern central Spain including mean, maximum, minimum, standard deviation, and prevalence values.

Parasite	Metric	All Animals	Infected Animals	Prevalence (%)
	Mean	19.44	46.12	42.15
GIN	SD	60.73	86.85	
	Min–Max	0–621	9–621	
	Mean	0.02	9.00	0.26
*T. discolor*	SD	0.47	–	
	Min–Max	0–9	9–9	
	Mean	1.26	3.25	38.74
*C. daubneyi*	SD	5.11	7.82	
	Min–Max	0–70	0.05–70	
	Mean	0.03	0.22	13.09
*F. hepatica*	SD	0.15	0.36	
	Min–Max	0–1.85	0.05–1.85	
	Mean	0.01	0.70	0.79
*D. dendriticum*	SD	0.10	1.13	
	Min–Max	0–2	0.05–2	
	Mean	0.16	63.00	0.26
*M. benedeni*	SD	3.22	–	
	Min–Max	0–63	63–63	
	Mean	12.95	77.27	16.75
*Eimeria* spp. ^1^	SD	74.85	169.80	
	Min–Max	0–1143	4.04–1143	
	Mean	0.09	6.56	1.31
*B. sulcata* ^1^	SD	1.06	7.07	
	Min–Max	0–6.4	1.65–18.75	

^1^ *Eimeria* spp. n = 362 and *B. sulcata* n = 352.

**Table 5:** Main parameters of fecal parasite counts (eggs, cysts and/or oocysts) of positive samples. *B. sulcata*, *D. dendriticum*, *M. benedeni*, and *T. discolor* prevalence were 1.31%, 0.79%, 0.26%, and 0.26%, respectively; with mean counts of 0.09, 0.01, 0.16, and 0.02 cyst eggs per gram, respectively.

	Prevalence (%)	Mean	SD	Min	Max	Spring	Summer	Autumn	Winter
GIN	42.15	19.44	60.73	9.00	621.00	23.20 ± 37.47	48.60 ± 97.23	106.55 ± 148.35	33.13 ± 53.17
*C. daubneyi*	38.74	1.26	5.11	0.05	70.00	3.71 ± 6.64	2.00 ± 1.00	3.31 ± 6.08	2.78 ± 9.91
*Eimeria* spp.	16.75	12.95	74.94	4.09	1143.00	30.21 ± 30.08	193.50 ± 357.10	122.56 ± 225.10	32.57 ± 37.16
*F. hepatica*	13.09	0.03	0.15	0.05	1.85	0.24 ± 0.45	0	0.11 ± 0.11	0.22 ± 0.36

**Table 6:** Co-infections with *C. daubneyi* in cattle fecal samples (n = 382) and farms (n = 40) surveyed in northwestern central Spain during the research. Percentages represent the proportion of samples and farms presenting each co-infection pattern.

Co-infections with *C. daubneyi*	Percentage of Samples	Percentage of Farms
GIN	9.69	37.50
*F. hepatica*	4.19	15.00
*Eimeria* spp. + GIN	2.62	10.00
*F. hepatica* + GIN	2.36	12.50
*Eimeria* spp.+ *F. hepatica*	1.57	10.00
*Eimeria* spp.	0.79	5.00
*Eimeria spp.* + *F. hepatica* + GIN	0.52	5.00
*B. sulcata*	0.52	2.50
*Eimeria* spp. + GIN+ *M. benedeni*	0.26	2.50
*D. dendriticum*	0.26	2.50
*D. dendriticum* + *F. hepatica* + GIN	0.26	2.50

**Table 7:** Seasonal prevalence (%) of main detected endoparasites in cattle, in northwestern central Spain during coprological survey by production system.

Season	Production System	GIN (%)	*C. daubneyi* (%)	*F. hepatica* (%)	*Eimeria* spp. (%)	Total (%)
autumn	Extensive	15	12.5	5	10	35
	Semi-extensive	2.5	2.5	0	0	5
spring	Extensive	15	17.5	10	0	25
	Semi-extensive	5	10	2.5	2.5	15
summer	Extensive	7.5	5	0	2.5	10
winter	Extensive	10	7.5	2.5	5	32.5
	Semi-extensive	10	5	0	2.5	17.5

**Table 8:** Statistical differences in fecal detection of identified endoparasites by season using Kruskal–Wallis and ANOVA tests.

Parasite	Kruskal–Wallis H	*p*-Value	Significant (KW)	ANOVA F	*p*-Value	Significant (ANOVA)
GIN	6.62	0.0849	No	6.44	0.0003	Yes
*C. daubneyi*	50.73	5.57 × 10^−11^	Yes	2.51	0.0587	No
*F. hepatica*	9.43	0.0240	Yes	1.20	0.3087	No
*Eimeria* spp.	21.63	7.78 × 10^−5^	Yes	5.29	0.0014	Yes

**Table 9:** Contingency table showing the coprological detection frequencies of *F. hepatica* and *C. daubneyi* in bovine fecal samples collected during the coprological survey.

	*C. daubneyi* Negative	*C. daubneyi* Positive
*F. hepatica* negative	217	115
*F. hepatica* positive	17	33

**Table 10:** Seasonal differences in parasite detection detected by Kruskal–Wallis and ANOVA tests. Significant differences were observed for *C. daubneyi* in spring, for GIN and *Eimeria* spp. in winter, and for *Eimeria* spp. also in autumn.

Parasite	Season	Kruskal–Wallis *p*-Value	ANOVA *p*-Value
*C. daubneyi*	spring	4.62 × 10^−11^	0.0113
*Eimeria* spp.	autumn winter	4.53 × 10^−6^ 0.0033	0.0003 0.0168
*GIN*	winter	0.0255	0.0192

## Data Availability

The data presented in this study are supported in the article. More detailed data is available on request from the corresponding author on reasonable request, due to ensure the privacy of farmers and livestock operations. Though the study complied with the General Data Protection Regulation (EU) 2016/679 [42].

## Supplementary Materials

The following supporting information can be downloaded at: https://www.mdpi.com/article/10.3390/pathogens14101057/s1, Table S1: Nematode egg output differences in different breeds. *AV= Avileña-Black Iberian cattle Negra Ibérica, LI = Limousine, CH = Charolais, FL = Fleickview, TU= Tudanca, MIX= mixed of several breed*; Table S2: Trematode egg output differences in different breeds. *AV = Avileña-Black Iberian cattle Negra Ibérica, LI = Limousine, CH = Charolais, FL = Fleickview, TU = Tudanca, MIX = mixed of several breed*; Table S3: Cestode faecal output differences in different breeds. *AV = Avileña-Black Iberian cattle Negra Ibérica, LI = Limousine, CH = Charolais, FL = Fleickview, TU = Tudanca, MIX = mixed of several breed*; Table S4: *Eimeria* spp. and *B. sulcata* faecal output differences in different breeds. *AV = Avileña-Black Iberian cattle Negra Ibérica, LI = Limousine, CH = Charolais, FL = Fleickview, TU = Tudanca, MIX = mixed of several breed*.
