# Supplementary material for: Evaluation of Gastrointestinal Endoparasites in Cattle in Central Spain: Focus on Calicophoron daubneyi with Coprological, Epidemiological, and Anthelmintic Insights
Source: Pathogens. 2025 Oct 19;14(10):1057. doi: 10.3390/pathogens14101057 (PMC12567455; doi:10.3390/pathogens14101057)
Supplement: Supplementary file 1 [file pathogens-14-01057-s001.zip › pathogens-3890497-supplementary.pdf]

pathogens

MDPI

Supplementary Materials:

Supplementary Tables S1, S2, S3, S4

Suppl. Table S1. A Student's *t*-test was performed to evaluate statistically significant differences in the shedding of GIN eggs among the cattle breeds present on the studied farms. The results obtained from the analysis are presented in the Table S1.

Table S1. Nematode egg output differences in different breeds. *AV=Avileña-Black Iberian cattle Negra Ibérica, LI= Limousine, CH= Charolais, FL= Fleickview, TU= Tudanca, MIX= mixed of several breed*

Breed	n	Mean	SD	Min	Max	Significant Comparisons
AV	92	12.8	41.1	0.0	369.0	
AV/LI	47	12.8	31.6	0.0	207.0	
CH	5	9.0	9.0	0.0	18.0	
FL	4	297.0	121.4	171.0	459.0	AV-FL (99%), CH-FL (95%), FL-LI (99%), FL-TU (99%), FL-AV/LI (99%), FL-MIX (99%)
LI	80	16.5	43.4	0.0	252.0	
MIX	144	21.4	69.7	0.0	621.0	
TU	10	1.8	3.8	0.0	9.0	CH-TU (95%)

Suppl. Table S2. A Student's *t*-test was performed to evaluate statistically significant differences in the shedding of trematode eggs among the cattle breeds present on the studied farms. The results obtained from the analysis are presented in the Table S2.

Table S2. Trematode egg output differences in different breeds. *AV=Avileña-Black Iberian cattle Negra Ibérica, LI= Limousine, CH= Charolais, FL= Fleickview, TU= Tudanca, MIX= mixed of several breed*

Breed	n	Mean	SD	Min	Max	Significant Comparisons
AV	92	0.4	3.1	0.0	30.0	
AV/LI	47	1.5	5.3	0.0	28.7	
CH	5	0.0	0.0	0.0	0.1	
FL	4	0.0	0.0	0.0	0.0	
LI	80	1.2	2.6	0.0	13.2	
MIX	144	1.7	6.8	0.0	70.0	AV-MIX (90%), AV-LI (90%)

TU	10	3.6	7.4	0.0	23.0	TU-AV (95%), TU-LI (95%)

Suppl. Table S3. A Student's *t*-test was performed to evaluate statistically significant differences in the shedding of cestode eggs among the cattle breeds present on the studied farms. The results obtained from the analysis are presented in the Table S3.

Table S3. Cestode faecal output differences in different breeds. *AV=Avileña-Black Iberian cattle Negra Ibérica, LI= Limousine, CH= Charolais, FL= Fleickview, TU= Tudanca, MIX= mixed of several breed*

Breed	n	Mean	SD	Min	Max	Significant Comparisons
AV	92	0.0	0.0	0.0	0.0	
AV/LI	47	1.3	9.2	0.0	63.0	AV/LI-MIX (90%)
CH	5	0.0	0.0	0.0	0.0	
FL	4	0.0	0.0	0.0	0.0	
LI	80	0.0	0.0	0.0	0.0	
MIX	144	0.0	0.0	0.0	0.0	
TU	10	0.0	0.0	0.0	0.0	

Suppl. Table S4. A Student's *t*-test was performed to evaluate statistically significant differences in the shedding of *Eimeria* spp. oocyts among the cattle breeds present on the studied farms. The results obtained from the analysis are presented in the Table S4.

Table S4. *Eimeria* spp. and *B. sulcata* faecal output differences in different breeds. *AV=Avileña-Black Iberian cattle Negra Ibérica, LI= Limousine, CH= Charolais, FL= Fleickview, TU= Tudanca, MIX= mixed of several breed*

Breed	n	*Eimeria* spp. Mean	*Eimeria* spp. SD	*Eimeria* spp. Max	*B. sulcata* Mean	*B. sulcata* SD	*B. sulcata* Max	Significant Comparisons
AV	92	8.6	31.7	189.0	0.0	0.0	0.0	
AV/LI	47	32.0	166.9	1143.0	0.2	1.1	6.4	
CH	5	0.0	0.0	0.0	0.0	0.0	0.0	
FL	4	0.0	0.0	0.0	0.0	0.0	0.0	
LI	80	8.8	26.5	162.0	0.0	0.2	1.7	
MIX	143	8.8	62.4	729.0	0.17	1.60	18.8	TU-MIX (95%), TU-LI (99%), TU-AV (99%)
TU	10	69.3	104.4	288.0	0.00	0.00	0.0	

Supplementary File S5. Epidemiological survey

File S5. Epidemiological survey was designed to collect detailed information from cattle farms regarding herd composition, age and breed distribution, management practices, and parasite control

strategies. The survey aims to identify potential risk factors associated with gastrointestinal parasitism and to support the interpretation of coprological and epidemiological findings within the study.

Farm Data

- Name of the farm: ____________________________
  Other comments or observations: ____________________________
- Location: ____________________________
  Other comments or observations: ____________________________
- Animal species present (cattle / sheep / dogs / cats / others): ____________________________
  Other comments or observations: ____________________________
- Productive purpose: ____________________________
  Other comments or observations: ____________________________

Animal Data

- Total number of animals by age and sex: ____________________________
  Other comments or observations: ____________________________
- Breeds present (name and number by sex): ____________________________
  Other comments or observations: ____________________________
- Age group separation (Yes / No): ____________________________
  Other comments or observations: ____________________________
- Separation of suckling calves with mothers from other young animals (Yes / No): ____________________________
  Other comments or observations: ____________________________
- If yes, until what age?: ____________________________
  Other comments or observations: ____________________________

Production System

- Production system (Extensive / Intensive / Semi-extensive / Other): ____________________________
  Other comments or observations: ____________________________
- Pasture rotation (Yes / No): ____________________________
  Other comments or observations: ____________________________
- Summer season (Irrigated / Rainfed): ____________________________
  Other comments or observations: ____________________________
- Type of production (Meat / Milk / By-products): ____________________________
  Other comments or observations: ____________________________
- Production cycle (Complete / Partial): ____________________________
  Other comments or observations: ____________________________
- Shared pasture with other species (Yes / No): ____________________________
  Other comments or observations: ____________________________

Deworming

- Number of annual deworming and active ingredients used: ____________________________

Other comments or observations: ____________________________

- Deworming season (Spring / Summer / Autumn / Winter): ____________________________

Other comments or observations: ____________________________

- Date and active ingredient of last deworming: ____________________________

Other comments or observations: ____________________________

- Use of cestocides (Yes / No): ____________________________

Other comments or observations: ____________________________

- Fasciola control (Yes / No): ____________________________

Other comments or observations: ____________________________

- Reason for choosing active ingredient (Veterinarian / Farmer / Price): ____________________________

Other comments or observations: ____________________________

- Coprological analysis prior to treatment (Yes / No): ____________________________

Other comments or observations: ____________________________
